# Legius Syndrome: two novel mutations in the *SPRED1* gene

**DOI:** 10.1038/hgv.2015.51

**Published:** 2015-12-03

**Authors:** Marika Bianchi, Veronica Saletti, Roberto Micheli, Silvia Esposito, Anna Molinaro, Stella Gagliardi, Simona Orcesi, Cristina Cereda

**Affiliations:** 1 Laboratory of Experimental Neurobiology, ‘C. Mondino’ National Neurological Institute, Pavia, Italy; 2 Developmental Neurology Unit, ‘C. Besta’ National Neurological Institute Foundation, IRCCS, Milan, Italy; 3 Child Neurology and Psychiatry Unit, Spedali Civili, Brescia, Italy; 4 School in Reproductive and Developmental Sciences, University of Trieste, Trieste, Italy; 5 Child Neurology and Psychiatry Unit, ‘C. Mondino’ National Neurological Institute, Pavia, Italy

## Abstract

The *SPRED1* gene encodes a protein involved in the Ras/MAPK (mitogen-activated protein kinase) signaling pathway. Mutations in *SPRED1* have been reported to cause Legius Syndrome, a rare developmental disorder that shares some clinical features with Neurofibromatosis-1. Direct sequencing was used to define *SPRED1* mutations. We present two previously undescribed mutations: a frameshift mutation causing a stop codon, which was identified in an Italian family (p.Ile60Tyrfs*18) and a missense variation, which was identified in one sporadic Italian case (p.Pro422Arg). Our results led us to hypothesize that these modifications may contribute to the Legius Syndrome phenotype. Further studies will be needed to determine the roles of these mutations in the mechanisms of Legius Syndrome.

The *SPRED1* gene, located on chromosome 15q13.2, encodes a protein of 444 amino acids that contains an N-terminal Enabled/VASP homology-1 (EVH-1) domain, a central KIT-binding domain (KBD) and a C-terminal SPRY domain.^1,2^ SPRED1 inhibits the Ras/Raf/MEK/ERK pathway as a negative growth factor, and is regulated by cytokine and chemokine-induced ERK.^2–4^

Heterozygous germline loss-of-function *SPRED1* mutations have been described in patients affected by Legius Syndrome,^5^ which is a developmental disorder that shares the pigmentary phenotype of and some additional clinical features with Neurofibromatosis-1 (NF1).^6^ Both of these syndromes belong to the group of rasopathies or neuro-cardio-facial-cutaneous syndromes, which are caused by germline mutations that affect proteins involved in the Ras/MAPK pathway.^3^

Legius Syndrome presents as an autosomal dominant condition characterized by multiple café-au-lait macules and skin fold freckling, with or without macrocephaly, a Noonan-like appearance, learning difficulties and/or attention deficit in children and by lipomas in adults. Some typical NF1 features, such as Lisch nodules of the iris, neurofibromas and central nervous system tumors are absent. Whether Legius Syndrome is associated with an increased risk for a specific range of malignancies remains unknown. Several *SPRED1* variants, including sequence-based changes and large deletions/duplications, have been linked to Legius Syndrome.^7,8^

All identified mutations in *SPRED1* are reported in the Leiden Open Variation Database (http://www.lovd.nl/SPRED1) and the Disease Databases on the ARUP Scientific Resource for Research and Education webpage (http://www.arup.utah.edu/database/SPRED1/SPRED1_welcome.php).

Here, we describe two novel mutations (p.Ile60Tyrfs*18 and p.Pro422Arg) in an Italian family with Legius Syndrome and in a sporadic case of Legius Syndrome.

Case 1: The family has four affected members: two 3-year-old probands who are bichorionic-biamniotic twins, their 35-year-old mother and their 66-year-old grandmother.

All individuals exhibited a similar phenotype: multiple café-au-lait spots and axillary freckling without macrocephaly and Noonan-like traits.

Dermatologic and ophthalmologic evaluations excluded lipomas, cutaneous neurofibromas and Lisch nodules in all of the family members. Brain magnetic resonance imaging (MRI) and cognitive assessments were performed in the twins, revealing normal results and mild language development delay.

Case 2: The proband was a 10-year-old boy who was referred to our department at 1 year of age. He was the first child of non-consanguineous Caucasian parents. His family history was unremarkable. On clinical examination, he exhibited multiple cafè-au-lait spots and bilateral axillary freckling. Macrocephaly and Noonan-like traits were not observed. Dermatologic and ophthalmologic evaluations, and abdominal sonography and brain MRI did not reveal any abnormal findings. His neuropsychological assessment was normal. The patient had shown vocal and motor tics since the age of 7, without any changes in behavior or scholastic performance and without features of an associated obsessive-compulsive disorder or attention deficit hyperactivity disorder.

Both cases were also tested for the *NF1* gene. The control subjects were recruited at the Transfusional Service and Centre of Transplantation Immunology, Foundation San Matteo’, IRCCS, Pavia, Italy.

All patients consented to genetic testing. The study was approved by the ‘C. Mondino’ Institute Review Boards and was performed in accordance with the Ethical Standards of the Declaration of Helsinki.

Genomic DNA was extracted from peripheral blood using an automated system (Maxwell 16 Blood DNA—Promega, Milan, Italy). Primer sequences are available on request.

All 7 coding exons of *SPRED1* were amplified and screened by direct sequencing using a Big-Dye Terminator v3.1 sequencing kit (Applied Biosystems, Milan, Italy) and an ABI 3130 Genetic Analyzer (Applied Biosystems). Each fragment was sequenced on both strands. The alignment to the reference sequence (NG_008980.1/NM.152594 RefSeqGene) was performed using Sequencer 4.8 software (Gene Codes Corporation, Ann Arbor, MI, USA).

The presence of genetic variants were verified on the NCBI, LOVD and Disease Databases on the ARUP Scientific Resource and were compared with data in the literature. The effects of the newly detected *SPRED1* mutations on protein structure and function were analyzed with the prediction programs ExPASy (http://www.expasy.org),^9^ PolyPhen (http://genetics.bwh.harvard.edu/pph/)^10^ and Project Hope software (http://www.cmbi.ru.nl/hope).^11^

Case 1: Both twins were heterozygous carriers of a novel frameshift mutation in exon 2 of the *SPRED1* gene (NM152594) c.367_1insT resulting in a stop codon (p.Ile60Tyrfs*18) (Figure 1). The insertion of the T nucleotide in exon 2 of the gene modifies the amino acid sequence of the SPRED1 protein, thus leading to a protein sequence that is shorter than the wild-type sequence, as predicted by the Translate tool from the ExPASy website. The mutant protein was predicted to be 78 amino acids. This mutation was also present in the mother and the grandmother. In addition, the asymptomatic members of the family were screened for *SPRED1*, and all were negative for the mutation.

Case 2: Sequencing results showed the presence of a *de novo* heterozygous variation in exon 7 of *SPRED1 (*NM152594) c.1024C>G. The mutation of CCT to CGT resulted in a substitution of proline to arginine in the protein at position 422 (p.Pro422Arg) (Figure 1). This residue is part of the Sprouty domain. Based on PolyPhen scores, this mutation is probably damaging to the protein (Hum Var 1/1 and HumDiv 1/1).

None of the identified mutations were found in 200 healthy Italian subjects sequenced for the *SPRED1* gene.

*SPRED1* encodes a protein that downregulates the Ras/MAPK pathway involved in cell growth and differentiation. Mutations in *SPRED1* cause Legius Syndrome, a condition characterized by intertriginous freckling, lipomas, macrocephaly, learning disabilities and developmental delay. Because the phenotype and some of the clinical features of Legius Syndrome are common in NF1 patients, the diagnosis of Legius Syndrome is difficult to make on the basis of clinical presentation alone, and genetic analysis is necessary to confirm the diagnosis and to exclude NF1 syndrome.

Several mutations that have been identified in the *SPRED1* gene lead to a non-functional or truncated protein that causes an increase in Ras/MAPK pathway activity.

Here, we report two novel mutations in an Italian family and in a sporadic case.

In the familial case, we identified a frameshift mutation that causes a premature stop codon. This truncated protein, which is smaller than the wild-type protein, presents an empty space in the EVH-1 protein domain.

In the sporadic case, a *de novo* missense mutation, p.Pro422Arg, in the Sprouty domain was identified. Due to their chemical properties, prolines are known to have a very rigid structure, sometimes forcing the protein backbone into a specific conformation. This *de novo* mutation introduces a proline residue that may cause a charge variation leading to the repulsion of ligands. Moreover, the hydrophobic interactions, either in the core of the protein or on the surface, would be lost in the mutant protein. Bioinformatics analysis led us to hypothesize that these modifications may cause Legius Syndrome.

However, further studies are required to assess the functional relevance of the detected variants and their potential association with the syndrome.

## Figures and Tables

**Figure 1:**
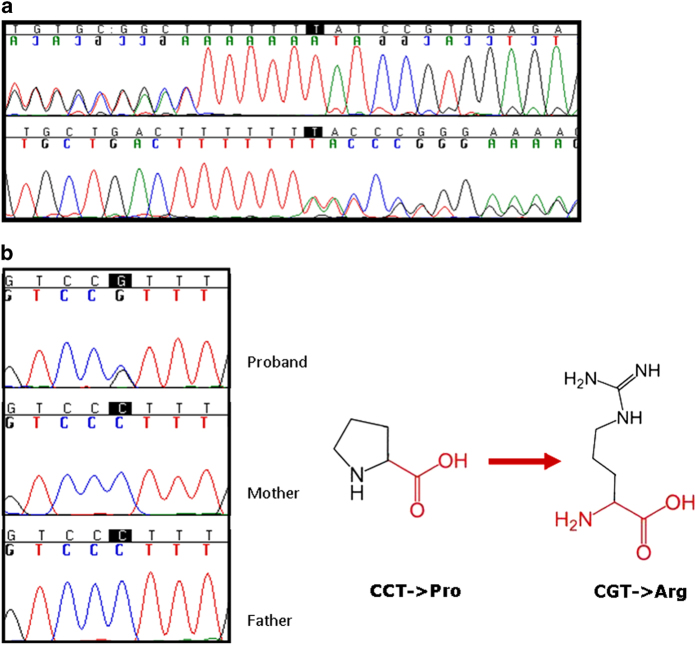
(**a**) The insertion found in the affected family is located in exon 2 and causes a stop codon (p.Ile60Tyrfs*18); (**b**) In the sporadic case, the missense mutation is located in exon 7 and causes an amino acid exchange (p.P422R).
